# Supplementary material for: Versatile Approach of Silicon Nanofabrication without Resists: Helium Ion-Bombardment Enhanced Etching
Source: Nanomaterials (Basel). 2022 Sep 20;12(19):3269. doi: 10.3390/nano12193269 (PMC9565762; doi:10.3390/nano12193269)
Supplement: Supplementary file 1 [file nanomaterials-12-03269-s001.zip › nanomaterials-1861683-supplementary.pdf]

Supplementary Materials

Versatile Approach of Silicon Nanofabrication without Resists: Helium Ion-Bombardment Enhanced Etching

Xiaolei Wen, Lansheng Zhang, Feng Tian, Yang Xu and Huan Hu *

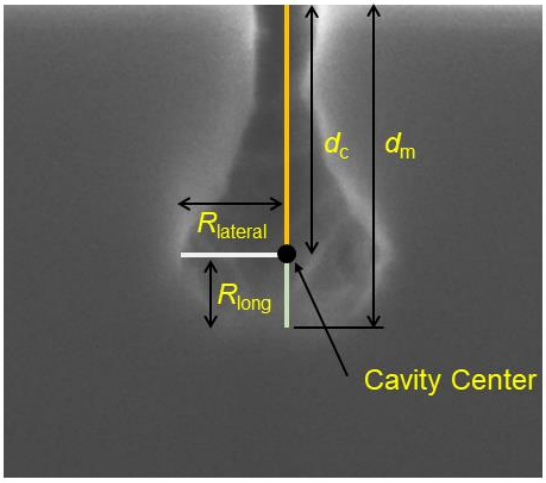

Figure S1. Measured parameters of the cavity: d_c is the vertical distance of the cavity center, d_m is the maximum depth of the cavity, $R_{lateral}$ is the lateral radius of the cavity, and R_{long} is the longitudinal radius of the cavity.

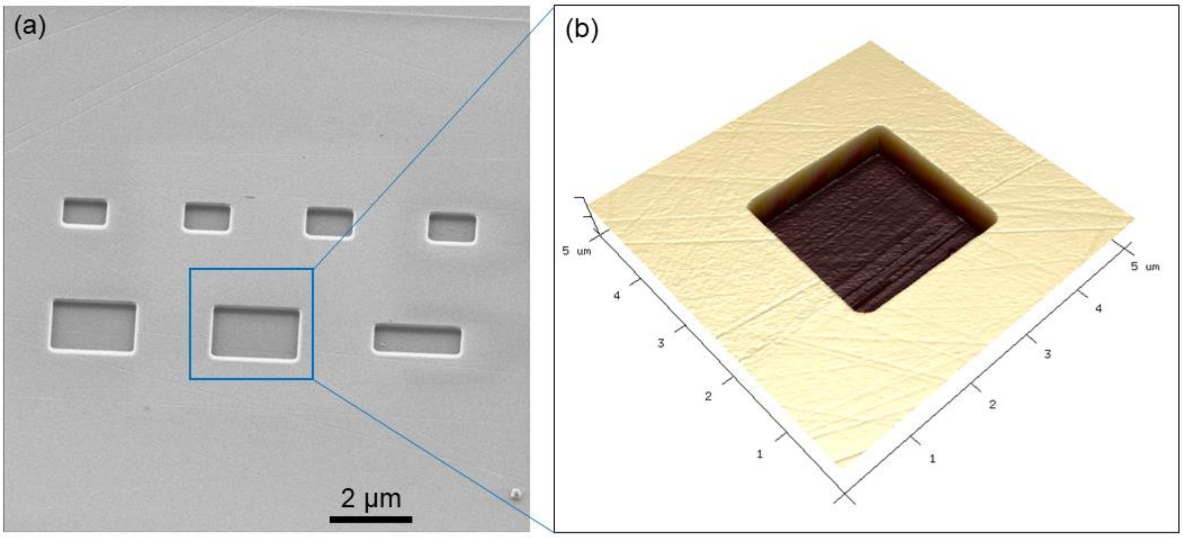

Figure S2. SiC micro-holes fabricated by HIBEE. (**a**) Helium ion microscopic image of micro-holes of SiC. (**b**) an AFM topography image showing that the depth of the hole is 217 nm and the surface roughness 0.753nm at the bottom surface of the hole.

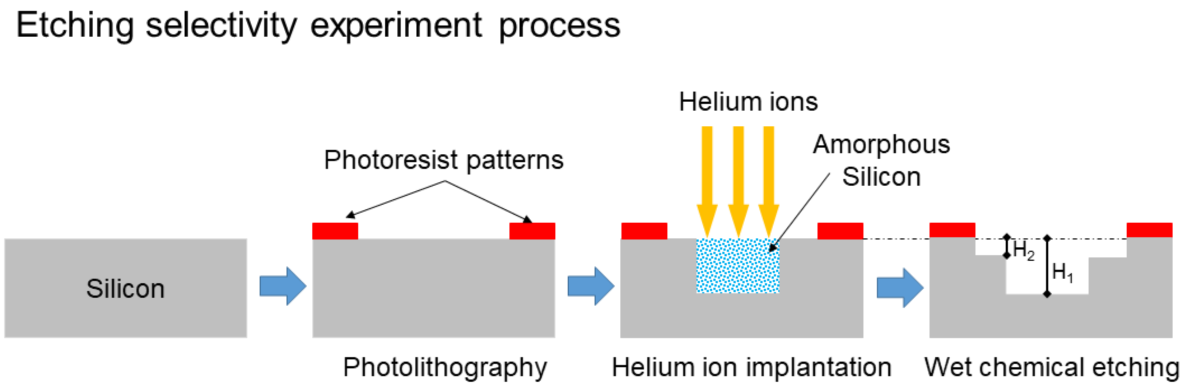

Figure S3. Schematics illustrating the process of the etching selectivity test.

The etching selectivity is given by H_1/H_2. H_1 is the etched depth for helium ion irradiated silicon and H_2 is the etched depth for non-irradiated silicon.

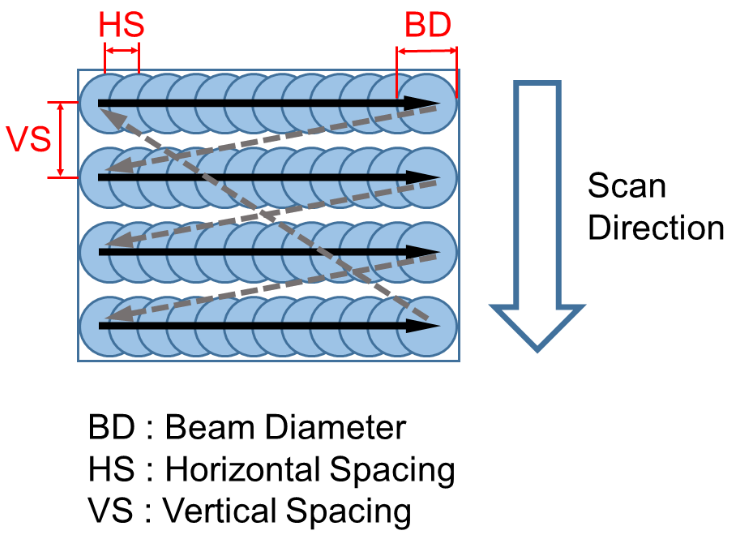

Figure S4. Schematic representation of helium ion dosing process.

The microscope hardware deflects the beam in a pre-determined pattern across the surface of the sample. A typical raster pass consists of deflecting the beam spot at fixed increments along one axis from one end to the other, dwelling for a given period at each point. At the end of the line, the beam skips down to the start point of the next line and moves the same way to the end. After finishing the whole frame of the pattern, the beam then returns to the very beginning of the first line and starts again. This process continues until all increments in the pattern reaches the desired dose. The beam spot diameter of the microscope is 0.5 nm, and the horizontal and vertical spacing between the increments in both axes is set to be 0.25 nm in this work.
